# Some Spinal Cord Lesions.—V

**Published:** 1911-08-26

**Authors:** 


### NEUROLOGY.

### SOME SPINAL CORD LESIONS.—V.

### Myelitis.

Local transverse myelitis, either acute or chronic, involving the cervical or dorsal regions of the cord, is followed by a descending degeneration of the pyramidal tracts below the lesion, and this leads to spastic paralysis of the legs. In acute myelitis paralysis and anæsthesia below the corresponding level of the lesion may develop in a day or two, or in some cases within a few hours. The earliest symptoms are malaise, tingling and numbness of the legs, pyrexia, hyperæsthesia, and twitching and cramps in the muscles. In subacute or chronic myelitis the onset is definite but much slower.

In a case of transverse myelitis affecting the mid-dorsal region there are paralysis and spastic rigidity of the legs; the arms are not affected. Loss of sensation in the legs and lower part of the body is bounded above by a narrow band of hyperæsthesia corresponding to the upper level of the lesion, which, if the middle dorsal segment were involved, would correspond to about the level of the ensiform cartilage. Exaggerated knee-jerks, ankle and patellar clonus and extensor plantar reflexes are present, while the elbow and wrist jerks remain normal.

There is no atrophy of the muscles of the legs except from disuse, and the arms and hands are normal. There is no reaction of degeneration. The sphincters are generally involved easily, so that involuntary passage of urine and fæces are prominent features, or involuntary passage of fæces and retention of urine with overflow.

The definite onset, the sensory disturbances, and the early impairment of the functions of the bladder and rectum distinguish this form of spastic paraplegia from primary spastic paraplegia and from amyotrophic lateral sclerosis, the latter being also excluded by the absence of wasting of the muscles of the hands.

In a case of local transverse myelitis of the lower cervical region, in addition to the above-mentioned symptoms found in transverse dorsal myelitis there would be: paresis or paralysis of the hands and arms; the band of hyperæsthesia is at a higher level; atrophy of the muscles of the hands and arms, similar to that seen in progressive muscular atrophy, amyotrophic lateral sclerosis, and syringomyelia, and leading by contracture to "main en griffe." Reaction of degeneration is given by the atrophied muscles of the hands.

High transverse myelitis is distinguished from amyotrophic lateral sclerosis by the definite or more or less acute onset, the sensory disturbances and the impairment of the functions of the bladder and rectum; from syringomyelia by the definite and more or less acute character of the onset, the loss of tactile sensibility as well as that of pain and temperature sense, and by the impairment of the functions of the bladder and rectum; and from progressive muscular atrophy both by the fact that there are disturbances of sensation and because the legs are affected as well as the hands.

### Tumours of the Spinal Cord.

Syphilitic, tuberculous, or gliomatous growths are the commonest forms of tumour that occur within the spinal canal; but even these are decidedly uncommon. Compression or destruction of the motor or sensory tracts and myelitis, with secondary degenerations, and, in the case of gliomata, cavitation, are the most important effects produced by the growth of a tumour in the cord.

The earliest symptoms are due to irritation, and there is intense burning or stabbing pain along the course of the nerves, which arise from the spinal cord at the level of the tumour. Pain may be increased by movement, but not to the same extent as when the tumour arises from the bone or meninges. In some cases paralysis is gradually produced without pain or spasm.

There may be progressive paralysis which at first is usually more marked in one leg than in the other. Ultimately there is muscular spasm of the legs and rigidity of the muscles of the back. If the cervical region is involved the arms may be paralysed as well as the legs, and the muscles of the neck may become stiff and contracted.

There may be hyperæsthesia of the skin at the level of the lesion and also a girdle pain. There may be impairment or total loss of all kinds of sensation in both legs. When one leg is more paralysed than the other the loss of sensation is sometimes said to be most marked in the limb which is least paralysed, but this is by no means a universal rule.

When the tumour is in the cervical or dorsal region the knee-jerks are exaggerated; there is ankle clonus, and the plantar reflexes are extensor in character. When the destructive lesion is in the lumbar enlargement or in the cauda equina, knee-jerks are diminished and finally lost, and there is a diminution or total loss of the plantar and cremasteric reflexes.

There is wasting of the muscles in the parts supplied by the nerve roots which are damaged. When the tumour is in the mid-dorsal region there is no wasting of the muscles of the legs except from disuse; but in the case of a tumour involving the lumbar region there may be wasting of the muscles of the feet and legs, and similarly as regards the hands and forearms if the tumour is in the cervical region.

There may be complete or incomplete reaction of degeneration in the atrophied muscles according to the extent to which the corresponding nerves are destroyed by the tumour.

There may be involuntary passage of urine and fæces or retention of urine with overflow.

Special sense organs are not, as a rule, affected.

